# The current state of training in psychiatry of intellectual disability: perspectives of trainees and trainers

**DOI:** 10.1192/bjb.2020.68

**Published:** 2021-02

**Authors:** Catherine Walton, Fionnuala Williams, Simon Bonell, Mary Barrett

**Affiliations:** 1 Swansea Bay University Health Board, UK; 2 Perth & Kinross Learning Disability Team, UK; 3 Plymouth Community Learning Disabilities Team, UK; 4 Leicestershire Partnership NHS Trust, UK

**Keywords:** Education and training, intellectual disability, recruitment and retention, learning disability, specialty training

## Abstract

**Aims and Method:**

Twelve intellectual disability psychiatry trainee representatives and 13 training programme directors were surveyed to assess the current state of training, to establish what motivated specialty trainees to choose intellectual disability psychiatry, and to explore issues that might affect retention.

**Results:**

The combined survey response rate was 83%. All trainees had chosen intellectual disability psychiatry after experience in either their personal or working life. Overall, specialty trainees were satisfied with their training; the majority felt supported to meet training requirements. Trainee isolation was the main concern for current trainees.

**Clinical implications:**

Recruitment for specialty training in intellectual disability psychiatry is acknowledged to be a concern for workforce planning and could affect access to and quality of psychiatric care for people with intellectual disability. The results of this survey could be used as a guide to improve efforts to attract trainees. Acknowledging and reducing trainee isolation could improve trainee morale.

People with intellectual disability have higher rates of mental health problems than the general population, estimated at between 30 and 50%.^1,2^ The complex interplay of predisposing biological, psychological and social risk factors for an individual with intellectual disability will have a significant effect on their presentation, which may not fit the ‘classic’ models of psychiatric presentations. Associated physical health problems such as epilepsy and sensory and communication problems can further complicate the presentation of psychiatric disorders. Social and environmental factors have a major role. Mental capacity, choice and control are also important issues to consider. These factors can make working in the specialty of psychiatry of intellectual disability challenging yet highly interesting and rewarding. Owing to clinical complexity, psychiatrists rarely work alone; there is a strong emphasis on multidisciplinary working and regular co-working with social care. However, psychiatrists may work as the sole doctor within a multidisciplinary team. These teams may be geographically distinct, at some distance from each other and based away from mainstream psychiatry colleagues.^3^ This can pose particular challenges for trainees in intellectual disability psychiatry, and there is evidence to suggest that trainees can feel isolated.^4^

Speciality training in psychiatry of intellectual disability in the UK comprises 3 years of training after attaining membership of the Royal College of Psychiatrists. This is in order to attain the Certificate of Completion of Training in Psychiatry of Learning Disability awarded by the General Medical Council (GMC). Trainees apply for a training post in a specific geographical area of the UK and usually rotate between a range of community and in-patient posts.

Recruitment in psychiatry in the UK has long been acknowledged as a concern in terms of workforce planning, with a lack of trainees recruited to fill gaps left by those retiring.^5^ Recruitment at the level of core training continues to be less than required – in 2012, 78% of year 1 posts were filled^6^ – and has remained static, with 78% of posts being filled in 2018.^7^ The ‘Choose Psychiatry’ campaign by the Royal College of Psychiatrists has aimed to improve recruitment to the specialty and successfully bolstered numbers of doctors entering core training by 30% between 2017 and 2018; however, the overall picture is that entry rates into the specialty remain similar to those of 2012.^8^

Once trainees have completed core training, they apply for specialty training posts. The fill rates for specialty training posts in psychiatry of intellectual disability do not compare favourably with those of other similarly sized subspecialties. Whereas fill rates have fluctuated for the other psychiatric subspecialties, recruitment has been especially poor in psychiatry of intellectual disability, where there have never been more than 30% of vacancies filled in each recruitment round since 2017.^9^ This is in spite of it scoring higher than general adult psychiatry (GAP), old age psychiatry, or child and adolescent psychiatry for overall trainee satisfaction in the 2019 GMC National Training Survey.^10^ Clearly, recruitment to the specialty is a challenge. It is predicted that there could be a shortfall in consultants in the specialty by 2033 of between four and 26 full-time-equivalent posts.^11^ Such data suggests a significant effect on the psychiatric care this vulnerable population will receive.

In order to inform future efforts to improve recruitment, we undertook a survey to investigate the reasons current trainees chose psychiatry of intellectual disability. We also assessed the current status of intellectual disability training in the UK by surveying the opinions of individuals currently in training and their trainers.

## Method

### Setting

This survey was aimed at trainers and trainees currently working in psychiatry of intellectual disability in the UK. All training programme directors (TPDs) and the network of Regional Trainee Representatives for psychiatry of intellectual disability were approached in order to obtain a spread of opinion across the four nations.

### Ethical considerations

An introduction to the survey gave potential respondents information regarding the aims and objectives of the survey. Implied consent was assumed by completion of the survey. The survey was created and agreed by the Specialty Advisory Committee of the Faculty of Psychiatry of Intellectual Disability.

### Data protection

Participants were approached using the email address provided to the Faculty. Once the survey link had been used, no further identifiable personal information was required.

### Survey design

A pilot survey of TPDs had been undertaken 1 year previously; the findings from this, along with information from the 2018 GMC Survey Specialty Specific Questions, were used to design the survey questions. The survey was created with SurveyMonkey and consisted of nominal and free-text questions. All questions requiring more than a ‘yes’ or ‘no’ response had free-text responses. Functionality and content validity were assessed by the authors, including both trainees and consultant psychiatrists.

The adaptive questionnaire design used two separate versions, depending upon whether the respondent identified as a trainee representative or as a TPD. Each version posed the same questions but from the perspective of either a trainee or a trainer. (The full survey is available in the supplementary material, available online at http://doi.org/10.1192/bjb.2020.68).

### Survey distribution

The survey link was emailed to the regional trainee representatives and TPDs in the specialty for each of the regions. The survey remained open for 6 weeks during early 2019. There was no incentive offered to complete the survey. A reminder email with a link was sent 1 week prior to the closure of the survey period.

### Response rate

There are 16 regions of the UK, with a total of 40 intellectual disability psychiatry specialty trainees, each represented locally by a trainee representative and a TPD. From the perspective of the trainee representatives, one region did not have an allocated representative, and one regional trainee representative was on long-term leave. Therefore, 12 of a possible 14 regional representatives responded, giving a trainee response rate of 86%. Of the TPDs, three individuals from the possible regions did not respond. The response rate for the trainers was 81%, giving a combined survey response of 83%. All returned surveys were fully completed with no missing answers.

### Analysis

All nominal responses were collated using the SurveyMonkey software. Primarily quantitative data were produced. The free-text answers were analysed separately by the four authors, and responses from the participants were used to illustrate the qualitative findings of the study.

## Results

### Respondent demographics

Of the trainee respondents, one was an ST4 trainee, two were ST5 and nine were ST6. For one area of the UK, there was no response from either the trainee representative or the TPD.

### Analysis of responses

#### Recruitment: the attraction of psychiatry of intellectual disability for a prospective trainee

##### Factors influencing choice of psychiatry of intellectual disability training

Trainees were asked about their core training experience. The majority of the trainees had completed a post in psychiatry of intellectual disability, or in both psychiatry of intellectual disability and child and adolescent mental health services (CAMHS). One trainee had completed neither. Trainees were asked to use free text to explain what factors had led them to choose higher training in psychiatry of intellectual disability. All 12 trainee representatives commented that their core training post had influenced their decision-making with respect to higher training options, from ‘opening their eyes’ to psychiatry of intellectual disability to ‘cementing a decision’. Other factors influencing their choice included seeking a good work–life balance and working within a multidisciplinary team. Medical school experiences were viewed as important, with one trainee having been influenced by a lecture given in medical school.

Personal experience outside medical training also had a strong influence for some respondents, with one trainee having worked with people with intellectual disability in a social care setting as a medical student, and another having a close family member with intellectual disability.

Fifty-four per cent of TPDs reported that core trainees in their area were required to undertake a developmental psychiatry post (CAMHS or intellectual disability) as part of their training, whereas 31% reported that this was not mandatory within the local training programme. The remainder were unsure. Currently, although competencies in developmental psychiatry are an important part of the core psychiatry curriculum and are best met through gaining experience in a developmental psychiatry post, this is not an essential training requirement.^12^

##### The role of dual training in improving recruitment

Dual training opportunities are now being considered as an option to improve the breadth of training, to meet the needs of a changing population and to improve recruitment. The only approved option currently available with psychiatry of intellectual disability is CAMHS, and very few training posts are advertised for this combination at present.

Trainees were asked whether they would have considered applying for dual training had it been available. Ten of the 12 trainees confirmed that they would have, with the majority opting for general adult psychiatry (GAP) or old age psychiatry. The reasons for choosing these subspecialties included the cross-over of cases, in particular, for individuals with mild or borderline intellectual disability in GAP, and those with dementia in old age psychiatry. More general reasons included a wider range of job opportunities for the future and an extension of training. From the perspective of trainers, 12 of the 13 TPDs responded that they would consider offering dual training posts. Most trainers also suggested considering dual training with forensic psychiatry. Other suggestions were GAP, old age psychiatry and CAMHS. Again, the reason cited for these choices was the overlap of the specialties. Dual training combining forensic psychiatry with intellectual disability was mentioned by one trainer as a need from the perspective of workforce planning in order to meet the requirements of the Transforming Care Programme.^13^

#### Retention: the current state of psychiatry of intellectual disability training in the UK

##### Special interest sessions

**Fig. 1:**
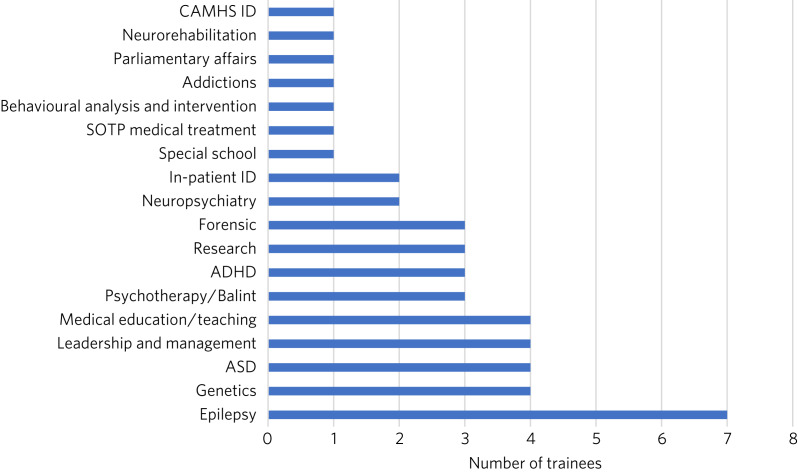
Special interest sessions. ADHD, attention-deficit hyperactivity disorder; ASD, autism spectrum disorder; ID, intellectual disability; SOTP, sex offender treatment programme.

Special interest sessions are an opportunity for trainees to broaden their perspectives and portfolio, to gain further experience and to understand other specialties allied to psychiatry of intellectual disability. Trainees should be able to spend up to a day each week on a special interest session or research of their choice. All trainees responded that their special interest sessions met their training needs. Figure 1 illustrates the breadth of options currently used by trainees.

Trainers responded that a wide range of special interest opportunities were available in their area or in neighbouring areas. According to trainees, barriers to accessing the sessions included a lack of time, conflict with other clinical commitments and difficulties travelling outside one's own trust for specific services.

##### Psychotherapy training

Half of the trainees surveyed responded that adequate supervised psychotherapy learning opportunities were available to them. Of those able to access these opportunities, 100% responded that the modalities available met their training curriculum needs.

The barriers to adequate opportunities, according to trainees, included a lack of supervision, with team psychologists often having their own students to supervise. Trainees sought clarity on the requirements for psychotherapy training, with a lack of formally agreed methods of supervision being cited as a barrier to accessing adequate experience.

The TPD responses were similar, with 46% responding that there was limited or no availability of psychotherapy opportunities available. Free-text responses mentioned the need for clarification of exactly what was required in terms of training needs for the intellectual disability population, and that a broad interpretation of what a psychotherapy learning opportunity entailed was required in order to allow a trainee to gain adequate experience.

##### Research

**Fig. 2:**
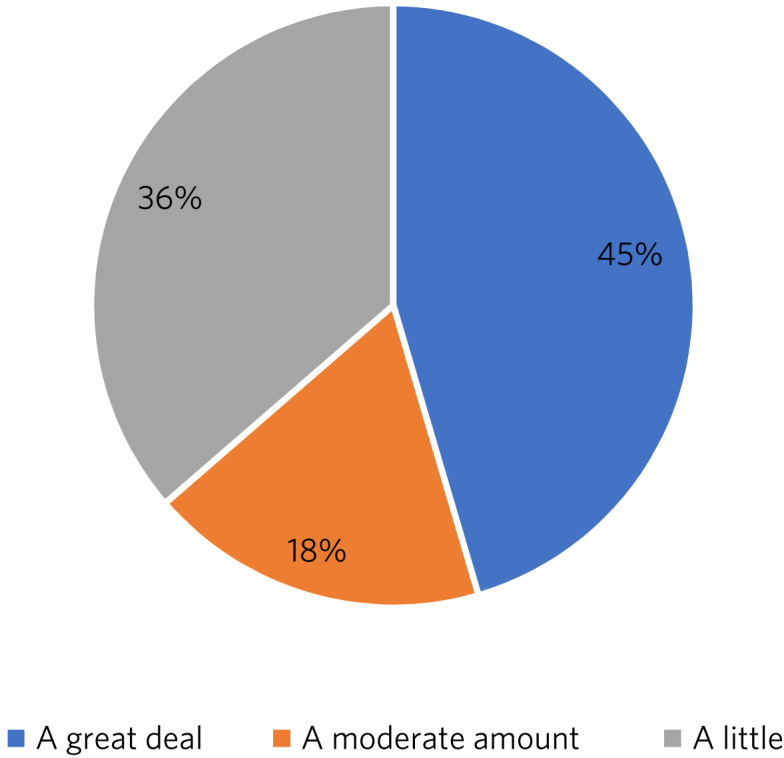
Trainee responses: are you adequately supported to carry out research?

As illustrated in Fig. 2, trainees were generally positive about accessing research opportunities in psychiatry of intellectual disability. Barriers cited by both trainees and trainers included a lack of protected time to undertake research and a clash with clinical commitments. Trainees found accessing research networks difficult, as well as knowing how to engage with an appropriate supervisor in the local area with specific intellectual disability research interests. It was acknowledged that it can be difficult to complete research projects during the 3 year training period. However, TPDs were very positive about research opportunities and all stated that they knew of research opportunities for trainees.

##### Clinical governance: audit and quality improvement

Responses indicated that audit networks have been established, and that 70% of trainees had a great deal or a lot of support to access these opportunities. Trainees reported that the consultant body tended to be experienced in this area and could offer support and project opportunities. Trainees also responded positively regarding access to quality improvement training, with 90% having lots of or moderate support. This appeared to be supported by pre-existing networks in place. However, with quality improvement being a relatively new entity, the lack of consultant experience in this area was cited as a barrier. This was reflected in the TPD responses, with two of the 13 (15%) respondents mentioning lack of experience and training of consultants in quality improvement as a barrier, along with a need for more clarity as regards training requirements in this area.

##### Out-of-hours experience

**Fig. 3:**
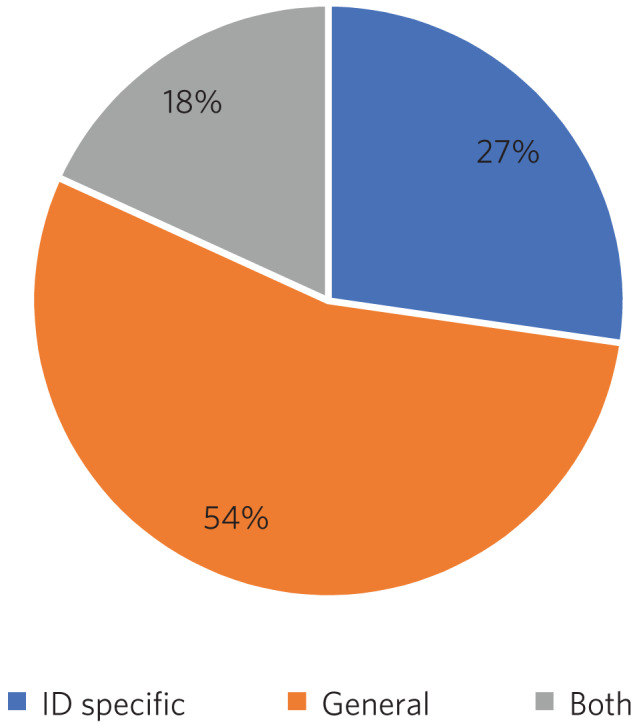
Psychiatry of intellectual disability (ID) out-of-hours experience.

Psychiatry of intellectual disability trainees are required to gain experience of emergency psychiatry, part of which includes being on an on-call rota for out-of-hours work. The rotas can vary regionally. As shown in Fig. 3, most trainees participated in a GAP rota; the trainees undertaking this rota found it a positive experience owing the opportunity to gain to increased emergency psychiatry and Mental Health Act experience as a trainee. There were some opposing opinions, however, with some trainees stating that the GAP rota did not give them enough out-of-hours experience in psychiatry of intellectual disability. Overall, trainees were positive, and out-of-hours work was found to meet training needs. The responses of the TPDs reflected those of the trainees; overall, they felt that the experience met training requirements.

##### Less than full time (LTFT) training

Of the 12 trainees, three were LTFT trainees. All replied positively regarding whether the current psychiatry of intellectual disability training programme supported their training needs. TPD responses were generally positive, indicating a belief that LTFT trainees receive adequate support to meet their training needs. However, it was noted that it can be more difficult for these trainees to access conferences and courses. One TPD stated that the support offered to LTFT trainees was a strength of the training scheme.

##### Retention: trainee well-being and support

All trainees responded that they felt supported in the training programme. Regular contact with approachable TPDs was cited as important. Trainees mentioned regular academic programmes and meetings with other trainees and clinicians as important aspects of trainee support. Supervisors who were available and approachable maintained this support.

One area has introduced a scheme allowing trainees to give feedback to neutral senior colleagues about training needs, which is then fed directly to the local TPD and Specialist Training Committee; this was reported by the local TPD to have been received positively.

Trainers and trainees both acknowledged trainee isolation; 30% of TPDs and 36% of trainees stated that they had experienced or noted trainee isolation personally. Reasons given for this were that psychiatry of intellectual disability is a small training scheme spread over wide geographical areas; therefore, in some areas, there are limited opportunities to meet with other trainees regularly. Solutions currently in place include a continuing professional development forum and digital solutions such as intellectual disability trainee WhatsApp groups. A lack of appointments to certain geographical regions, or trainees leaving posts, has also added to isolation in some regions.

## Discussion

The aim of this survey was to assess trainees’ reasons for choosing psychiatry of intellectual disability and to find out more about the current state of intellectual disability training in the UK. The survey had a good overall response rate, with 86% of regional trainee representatives and 81% TPDs responding.

In terms of choice of specialty, it was apparent that previous experience within the specialty was critical to the choice of the majority of trainees. In the main, this was core training experience, but trainees highlighted other experiences such as medical school lectures as having an effect. The influence of experience in working with individuals with intellectual disability on choice of future work has been demonstrated widely, including in other health services, for instance, in Australia.^14^ It is concerning, therefore, to find that at least one-third of areas do not currently require core trainees to undertake a clinical placement in developmental psychiatry. At present, a review of the curricula is being undertaken by the Royal College of Psychiatrists; therefore, there is potential for this to change in the future.

Recruitment strategies need to include lobbying for more core psychiatry trainees to have opportunities to rotate through psychiatry of intellectual disability. Forging links with medical schools and offering regular experiential and teaching opportunities would also raise the profile of psychiatry of intellectual disability. Current intellectual disability trainees have presented at the National Student Psychiatry Conference in order to improve knowledge of the specialty and access to further experiences.

It was apparent that current trainees were very interested in the option of dual training with another specialty to broaden training opportunities and experiences. This finding is in keeping with the findings of a recent survey of dual trainees in old age psychiatry and GAP^15^ and fits with recommendations from the Shape of Training review (2013),^16^ which aimed to broaden training experiences to meet changing patient requirements. The option of dual training is currently being explored by the Psychiatry of Intellectual Disability Specialist Advisory Committee as part of the curriculum rewrite process and should also be considered by the wider Faculty. However, the current system of advertising training numbers may be a barrier to offering further dual training opportunities. The system allows very little flexibility, which will need to be addressed.

Overall, trainees and trainers responded positively to questions about current training. All the trainees felt supported, including positive responses from LTFT trainees. Trainees felt supported to undertake special interest sessions and had undertaken a broad range of these. Responses to questions about research opportunities were more varied, with common barriers cited by both trainees and trainers; these were mainly due to a lack of protected time to undertake research. Psychotherapy training, where available, was reported to be of a quality such that trainees were able to meet the requirements of the curriculum, but it was clear that there were some regions where trainees were unable to access adequate supervision and support. Out-of-hours experience varied between regions, but overall trainees felt that this met their training needs.

A third of both groups reported trainee isolation. Reduced recruitment to training posts in certain regions and trainees leaving the specialty were among the reasons given for this. Trainee isolation has been reported previously in the literature,^4^ and physical isolation can be further compounded by feelings of stigma by association – the process by which relatives, friends, support staff and associates feel stigmatised by contact with a stigmatised or marginalised group, such as those caring for a vulnerable patient group with intellectual disability who face marginalisation and disadvantage in their daily lives.^3^ Acknowledgment of this as an issue continues and there is ongoing work in this area. Psychiatry of intellectual disability trainees are now invited to join ‘Basecamp’, an online forum where trainees can communicate with each other, ask questions and raise concerns. It is managed by the national trainee representatives who meet regularly with the Faculty of Psychiatry Intellectual Disability Executive Committee and the Specialty Advisory Committee. On a more local level, regular meetings with TPDs, academic sessions and trainee networks have reduced trainee isolation and have received positive feedback locally. Trainee support groups for issues specific to intellectual disability trainees have worked well for geographical networks of trainees.^3^

In the current climate of political and economic uncertainty, recruitment to medicine in general is a challenge. Doctors are choosing to take longer breaks between foundation and specialty training,^17^ and recruitment to core training posts remains static.^6,7^ Psychiatry has traditionally been considered a less glamorous ‘Cinderella specialty’, losing out in recruitment to the larger medical specialties. As a small subspecialty, intellectual disability psychiatry loses out again among the psychiatric subspecialties.^9^ Investment in recruitment with campaigns such as ‘Choose Psychiatry’^18^ will go some way towards increasing awareness of the benefits of training in psychiatry in general. It is also hoped that the introduction of foundation fellowships^19^ will encourage high-quality trainees into psychiatry.

For intellectual disability psychiatry training specifically, there is further scope to highlight the results of the GMC National Training Survey data^7^ and overall trainee satisfaction rates, which reflect favourably on intellectual disability training. The positive results of this survey also highlight the benefits of intellectual disability training in terms of trainee support, scope for LTFT training, and flexibility in special interest sessions and research. Opportunities to experience intellectual disability psychiatry are widening and include an intellectual disability psychiatry taster programme that has been developed successfully in the West Midlands.^20^ The development of foundation programme posts in intellectual disability psychiatry could increase exposure to the specialty, and there is scope to broaden this further, with five foundation posts currently available in the UK. The 2019 National Intellectual Disability trainee conference, held in Cardiff, offered discounted entry for medical students and for foundation and core trainees. Such national events showcase the scope of opportunity within intellectual disability psychiatry and give opportunities for all, including medical students, to contribute posters and presentations, increasing audience participation and interest.

A strength of this survey was its good response rate, at 83%, with full completion of the returned questionnaires. The survey covered multiple geographical regions for both trainees and TPDs. There was one geographical region with no representation from either trainer or trainee. However, the accuracy and generalisability of the findings were limited by the low overall number of participants. The selection of only trainee representatives and TPDs could have led to bias, for example, toward selecting those trainees with a more positive training experience. The personal characteristics of a trainee representative could also cause bias, with such representatives potentially being more engaged and having more awareness of opportunities in their local area. TPDs may offer a better training experience to trainees on placement with them and therefore assume that all other posts in the respective deanery are also positive. Sending the survey out to all trainees was considered; however, when this has been attempted with similar surveys in the past, the response rate has been poor, and data protection requirements led to further complications. It was felt that targeting trainee representatives was likely to lead to a better response rate and a broader picture of training across the UK. The fact that certain regions did not have a response from both trainee and TPD could have biased results, with those regions potentially having empty posts or reflecting areas with more challenging training experiences or less engaged trainers. Broadening the scope of the survey to capture the views of core trainees who did not choose intellectual disability psychiatry, and the reasons why, would be of particular value for future recruitment.

## Conclusions

This survey of trainers and trainees across the UK indicates that, overall, intellectual disability trainees are broadly positive about their training and feel supported, with adequate training opportunities. Trainee isolation is a theme that has been highlighted and might be remedied by the improvement of trainee networks. The survey demonstrates that there is scope to continue improving training opportunities, in particular for psychotherapy and research. The opportunity for dual training was popular with the survey cohort; this is a potential key finding in terms of recruitment.

Trainees have provided insight into their reasons for choosing the subspecialty. Hopefully, this will guide improvements in recruitment to this rewarding subspecialty of psychiatry. The survey showed that a key motivator for trainees selecting this specialty was having had a core training placement in intellectual disability. Increasing the availability of such opportunities may not only help to bring people into the subspecialty, but also ensure that all trainees have a good grasp of intellectual disability psychiatry, which is important whatever specialty of psychiatry they ultimately choose. Recruitment to psychiatry is a continuing concern, with current and long-term impact on patient care to be considered. This survey contributes to a much broader picture that needs further research to investigate key motivators and barriers regarding choice of higher training specialties.

## About the authors

**Catherine Walton** is an ST6 in Psychiatry of Intellectual Disability, Swansea Bay University Health Board, Cardiff, UK. **Fionnuala Williams** is a Consultant in Psychiatry of Intellectual Disability, Perth & Kinross Learning Disability Team, Murray Royal Hospital, Perth, UK. **Simon Bonell** is a Consultant Psychiatrist for Adults with Learning Disabilities and Training Programme Director for Learning Disability Psychiatry, Peninsula Deanery, Plymouth Community Learning Disabilities Team, Westbourne, Plymouth, UK. **Mary Barrett** is a Consultant Psychiatrist for Adults with Learning Disabilities, East Midlands Region Training Programme Director for Psychiatry of Learning Disability, and Chair of the Psychiatry of Intellectual Disability Specialty Advisory Committee, Leicestershire Partnership NHS Trust, Learning Disability Service, Leicester, UK.

## Supplementary material

For supplementary material accompanying this paper visit https://doi.org/10.1192/bjb.2020.68. click here to view supplementary material
