# Supplementary material for: The current state of training in psychiatry of intellectual disability: perspectives of trainees and trainers
Source: BJPsych Bull. 2021 Feb;45(1):59–65. doi: 10.1192/bjb.2020.68 (PMC8058933; doi:10.1192/bjb.2020.68)
Supplement: Supplementary file 1 [file S2056469420000686sup001.zip › S2056469420000686sup001.docx]

**Intellectual disability trainee / trainer survey**

1. Are you a trainee or trainer?

**Trainees:**

1. What level of training are you at?
2. Region
3. Did you do a neurodevelopmental post as a core trainee?
4. How much did the placement influence your decision to do ID training? What other factors influenced your decision? (free text response)
5. Dual training opportunities are being considered as a way of improving the breadth of training, meeting the needs of a changing population and improving recruitment. Currently CAMHS/ID is the only approved option. Would you have considered applying for dual training? (yes / no). If yes – which subspecialties would appeal and why? (free text response)
6. Regarding Special Interest sessions: What sessions have you undertaken?
7. Regarding Special Interest Sessions: Did each meet your curriculum learning needs?
8. Regarding Special Interest Sessions: Are there any opportunities not available? Are there any barriers to obtaining special interest session experience?
9. Psychotherapy: Are supervised psychotherapy learning opportunities available on your rotation?
10. Psychotherapy: What modalities / opportunities have you had?
11. Psychotherapy: Did each meet your curriculum learning needs?
12. Psychotherapy: What barriers are there to obtaining psychotherapy experience?
13. Regarding research opportunities: Does your rotation adequately support you to carry out research?
14. Regarding clinical governance: Audit - Does your rotation adequately support you to carry out audit? Please specify support mechanisms or barriers (free text response)
15. Regarding clinical governance: Quality Improvement - Does your rotation adequately support you to carry out quality improvement work? Please specify support mechanisms or barriers.
16. Regarding clinical governance: Quality Improvement - Does your rotation adequately support you to carry out quality improvement work? Please specify support mechanisms or barriers.
17. Trainee Support: Do you feel supported as a trainee?

If yes, what do you find helpful? If no, what are the reasons for this? (free text response)

1. Trainee isolation: Do you ever feel isolated as a trainee?

If yes, what might help? If no, what support systems are you aware of? (free text response)

1. Out of Hours Experience. What experience do you get out of hours? (free text response)
2. Out of Hours Experience. Does the ID experience meet your training needs? If no, what is missing? (free text response)
3. Less than full time training. Do you work less than full time?

If yes, are you gaining the experience you require to meet your training needs (albeit over a longer period of time?) (free text response)

**Training Program Directors:**

1. Region
2. Choosing ID higher training – are core trainees required to do a neurodevelopmental post in local schemes?
3. Dual training opportunities are being considered as a way of improving the breadth of training, meeting the needs of a changing population and improving recruitment. Currently CAMHS/ID is the only approved option. Would you consider offering dual training posts? If yes, with which specialty / specialties and why? (free text response)
4. Regarding special interest session opportunities (Specify mentoring and leadership, psychotherapy, ASD, ADHD, epilepsy, genetics etc.). Are there any special interest opportunities not available in your rotation? What arrangements are in place for trainees to gain experience in these areas?
5. Psychotherapy Competencies. Are there any barriers to obtaining psychotherapy experience for trainees? Please give details.
6. What research support is in place for trainees? Are there any barriers?
7. Regarding clinical governance: Audit - What audit support is in place for trainees? Are there any barriers?
8. Regarding clinical governance: Quality Improvement What quality improvement support is in place for trainees? Are there any barriers?
9. Do your trainees feel supported? What support structures are in place? If you think they do not feel supported why is this?
10. Do your trainees ever report feeling isolated? If yes, why might this be? What is available to help reduce isolation?
11. Out of hours experience: what on-call experience do your trainees get? (ID specific, general adult psychiatry or both). Does it meet their training needs? If not, what is missing?
12. Less Than Full Time (LTFT) Training Do you feel the training programme lends itself to supporting LTFT trainees personal and professional needs? Are there any barriers to this?
